# Automated *in vivo* drug screen in zebrafish identifies synapse-stabilising drugs with relevance to spinal muscular atrophy

**DOI:** 10.1242/dmm.047761

**Published:** 2021-04-26

**Authors:** Ana-Maria Opris¸oreanu, Hannah L. Smith, Sophia Krix, Helena Chaytow, Neil O. Carragher, Thomas H. Gillingwater, Catherina G. Becker, Thomas Becker

**Affiliations:** 1 Centre for Discovery Brain Sciences, University of Edinburgh, The Chancellor's Building, 49 Little France Crescent, Edinburgh EH16 4SB; 2 Euan MacDonald Centre for Motor Neurone Disease Research, University of Edinburgh, EH16 4SB Edinburgh, UK; 3 Cancer Research UK Edinburgh Centre, MRC Institute of Genetics and Molecular Medicine, University of Edinburgh, EH4 2XR Edinburgh, UK

**Keywords:** Drug discovery, Phenotypic screening, Chondrolectin, VAST, Zebrafish, Synapse stabilization

## Abstract

Synapses are particularly vulnerable in many neurodegenerative diseases and often the first to degenerate, for example in the motor neuron disease spinal muscular atrophy (SMA). Compounds that can counteract synaptic destabilisation are rare. Here, we describe an automated screening paradigm in zebrafish for small-molecule compounds that stabilize the neuromuscular synapse *in vivo*. We make use of a mutant for the axonal C-type lectin *chondrolectin *(*chodl*), one of the main genes dysregulated in SMA. In *chodl−/−* mutants, neuromuscular synapses that are formed at the first synaptic site by growing axons are not fully mature, causing axons to stall, thereby impeding further axon growth beyond that synaptic site. This makes axon length a convenient read-out for synapse stability. We screened 982 small-molecule compounds in *chodl chodl−/−* mutants and found four that strongly rescued motor axon length. Aberrant presynaptic neuromuscular synapse morphology was also corrected. The most-effective compound, the adenosine uptake inhibitor drug dipyridamole, also rescued axon growth defects in the UBA1-dependent zebrafish model of SMA. Hence, we describe an automated screening pipeline that can detect compounds with relevance to SMA. This versatile platform can be used for drug and genetic screens, with wider relevance to synapse formation and stabilisation.

## Abstract (precis)

**Summary:** We report an automated and high-throughput screening platform to identify compounds relevant to motor neuron disease using younger, less-pigmented zebrafish embryos.

## INTRODUCTION

Zebrafish (*Danio rerio*) embryos are versatile models to investigate the mechanisms of motor neuron diseases owing to the relative ease with which one can analyse the morphology and growth of the motor axons (reviewed by Babin et al., 2014; Pappalardo et al., 2013; Patten et al., 2014). They are also a suitable tool to perform cost-effective chemical library screenings of existing drugs or novel small-molecule compounds with pharmaceutical potential *in vivo* (Corman et al., 2019; McGown et al., 2016).

Neuromuscular innervation in zebrafish embryos is well characterized, with motor neurons classified into primary and secondary motor neurons, based on their time of differentiation and of muscle innervation. Mainly, there are three primary motor neurons per trunk hemisegment: caudal primary (CaP), middle primary (MiP) and rostral primary (RoP). A variably present motor neuron (VaP) is present in 50% of the hemisegments. The primary motor neurons begin to differentiate ∼9 h post fertilisation (hpf); at 16 hpf their axons grow out ventrally onto a common pathway to the horizontal myoseptum (HM), an intermediate target, where axons will pause and – with the so-called muscle pioneers – make synapses. Muscle pioneers are the first cells to display muscle-specific characteristics (Felsenfeld et al., 1991; Hatta et al., 1991; Myers et al., 1986). Recent evidence shows that formation of these ‘en passant’ synapses (i.e. synapses formed during axon growth along the trajectory of an axon, which are not located at the axon terminal) between motor axons and muscle pioneer cells may be necessary for subsequent motor axon growth (Opris¸oreanu et al., 2019). From here, axons diverge into their paths with CaP axons growing ventrally, MiP axons extending dorsally and RoP axons growing laterally along the trunk. Secondary motor axons follow the trajectories of the primary motor neurons (Beattie et al., 2002; Myers et al., 1986; Seredick et al., 2012; Westerfield et al., 1986).

Manipulation of gene expression related to human motor neuron diseases often cause changes in the stereotypical appearance of the CaP motor axons in zebrafish embryos. For example, in the zebrafish model of amyotrophic lateral sclerosis (ALS), overexpression of mutant SOD1 leads to aberrant branching and shortening of the primary motor axons (Lemmens et al., 2007), whereas knockdown of C9orf72, associated with ALS, causes aberrant branching of CaP motor axons (Ciura et al., 2013). In different genetic zebrafish models of spinal muscular atrophy (SMA) targeting the *smn* gene, aberrant axonal branching and disruption of CaP motor axon outgrowth has been observed (Hao et al., 2011, 2013; McWhorter et al., 2003). Another established model phenocopying the SMA-related motor neuron morphology is that of the ubiquitin-like modifier activating enzyme 1 (UBA1). Inhibition of the Smn-binding partner UBA1 by the cell-permeable ubiquitin E1 inhibitor UBEI-41 (also known as Pyr-41) disrupts CaP motor axon outgrowth and leads to abnormal axon morphology in zebrafish. Dysregulation of the UBA1 pathway is associated with accumulation of β-catenin, and inhibition of β-catenin signalling by quercetin reverts the UBA1-dependent neuromuscular pathology in zebrafish, *Drosophila* and mouse models of SMA (Wishart et al., 2014).

In SMA, motor neurons degenerate owing to loss of expression of the *SMN1* gene, which is involved in RNA splicing. Hence, loss of SMN1 protein leads to downstream gene dysregulation. In SMA mouse models, one of the earliest genes dysregulated is *chondrolectin *(*Chodl*) (Bäumer et al., 2009; Zhang et al., 2008). Chodl is an axonal type I transmembrane C-type lectin protein (Weng et al., 2002, 2003), expressed by zebrafish motor neurons (Zhong et al., 2012) and mouse ‘fast’ motor neurons (Enjin et al., 2010; Wootz et al., 2010). Chodl has a conserved role in neuromuscular synapse differentiation in both mice and zebrafish, and in subsequent synapse-dependent motor axon growth in zebrafish (Opris¸oreanu et al., 2019). In zebrafish, *chodl* is necessary for both growth of primary motor axons beyond the HM synaptic site and later branching of motor axons in a cell-autonomous fashion for motor neurons. Knockdown or knockout of *chodl* in zebrafish induces prolonged stalling of the CaP motor axons at the HM, probably due to impaired synaptogenesis (Opris¸oreanu et al., 2019; Zhong et al., 2012). The neuromuscular synapse destabilization observed in the early phase of SMA disease progression (McGovern et al., 2008) could, thus, involve dysregulation of *chodl* (Bäumer et al., 2009; Zhang et al., 2008). Importantly, the axonal phenotype in the zebrafish model of SMA is partially rescued by overexpression of *chodl* (Sleigh et al., 2014), suggesting that *chodl* function is a key factor in the disease mechanism. Aberrant processing of *Chodl* mRNA has also been found in a mouse model of ALS (*SOD1^G93A^*) (Wootz et al., 2010). Therefore, to identify small-molecule compounds that stabilize synapses in the absence of *chodl* in zebrafish could be relevant in the discovery of new therapeutics, not only for SMA but also for ALS or other conditions comprising destabilising synapses.

Here, we combined the use of the zebrafish *chodl−/−* mutant (hereafter referred to as *chodl* mutant) with a semi-automated chemical library screening platform to screen for synapse-stabilization compounds. We used axon length as a simple and quantifiable read-out with subsequent direct observation of synapse morphology in the UBA1 model of SMA. We screened 982 small molecules on the *chodl* mutant and identified four compounds that were able to rescue the axonal length phenotype. Two drugs, IOX1 and dipyridamole, rescued the presynaptic defects in the *chodl* mutant and, of these, dipyridamole also rescued the axonal growth defects in the UBA1 model of SMA, indicating that our screening approach can identify synapse-stabilising compounds with potential relevance to SMA.

## RESULTS

### Establishment of a chemical genetic screen for small-molecule compounds that stabilize the neuromuscular synapse in zebrafish

We performed an automated drug screen of 982 small molecules (ENZO kinase, epigenetic and protease inhibitors libraries, SGC Epigenetic Probe Collection, Enzo FDA-approved drug library V2) by using a zebrafish *chodl* mutant line, in which motor neurons are transgenically labelled (*chodl−/−; mnx1:EGFP*). To automate the screening process, we used a Large Particle (LP) Sampler and VAST BioImager coupled to a spinning disk confocal microscope (Early et al., 2018; Pardo-Martin et al., 2010). This system uses pigmentation patterns to orient specimen and, therefore, had previously only been described to accept older embryos and larvae (48, 72 and 96 hpf) (Ansar et al., 2019; Early et al., 2018; Pardo-Martin et al., 2010). In our study, we adapted the protocol for the VAST BioImager for the use of 28 hpf embryos, refining several parameters in order to automatically load, detect and image the weaker pigmented younger embryos. Changed parameters, pertaining to pump settings, load/unload settings and object detection setting (see Materials and Methods), allowed for successful automatic processing of 28 hpf embryos when using the VAST BioImager.

**Fig. 1.**
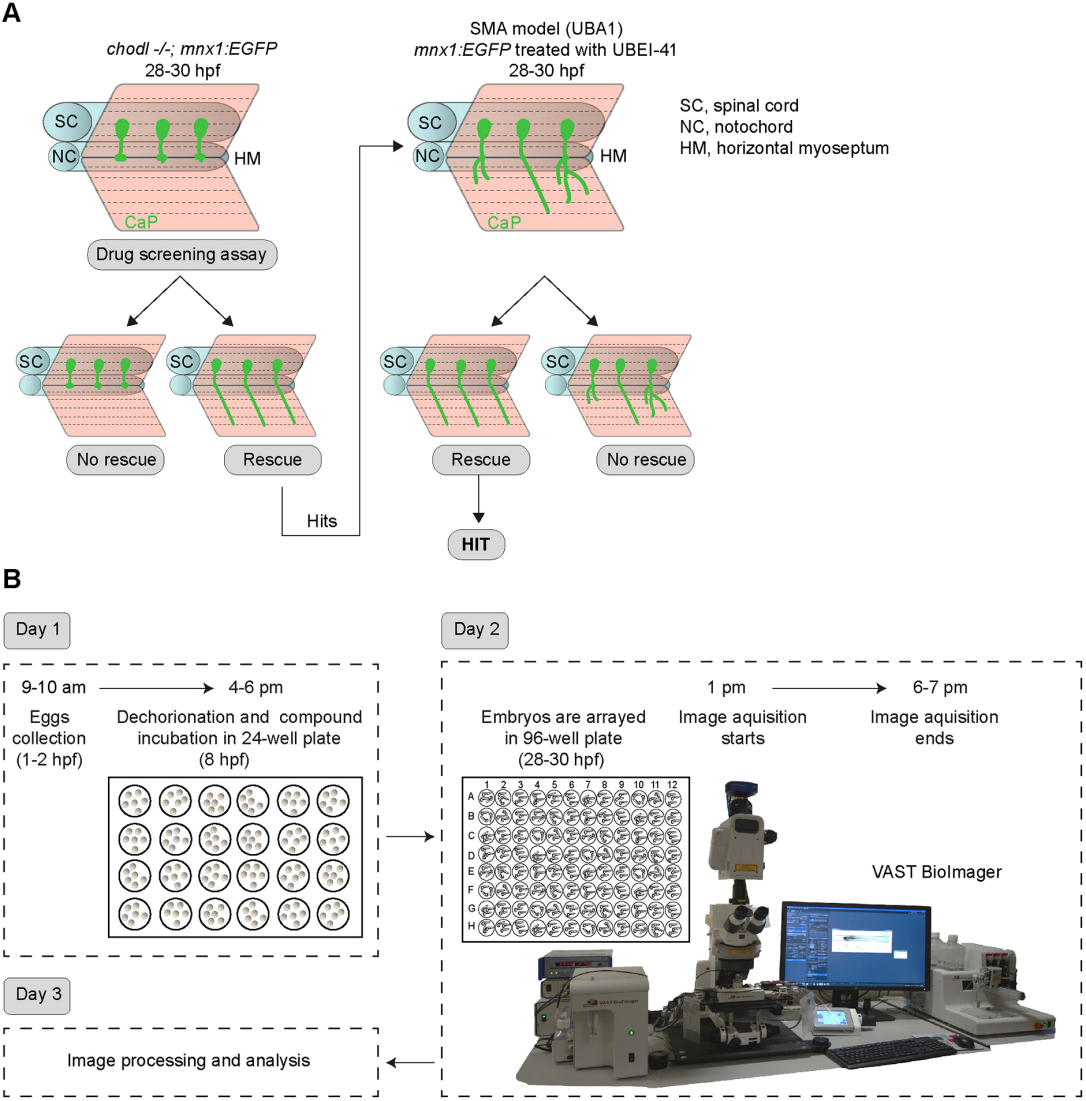
**Automated small-molecule compound screening follows a two-step workflow.** (A) Schematic representation of the axonal phenotype in *chodl−/−; mnx1:*GFP embryos (left) with stalled axons at the horizontal myoseptum (HM), and zebrafish model of SMA (UBA1 model, right) showing abnormal motor axons, as used in drug screening. Chemical compounds that rescue the *chodl−/−; mnx1:EGFP* axonal phenotype are tested again using the UBA1 model. SC, spinal cord; NC, notochord. (B) Schematic representation of timeline and workflow of the experimental protocol. At 8 hpf, zebrafish eggs are arrayed in a 24-well plate (six eggs per well) and incubated in drug solution overnight. The next day (day 2) the 28-30 hpf embryos are moved from the 24-well plate into a 96-well plate (three embryos per well) followed by automated imaging using the VAST BioImager. In a single imaging session, between 16 and 20 chemical compounds can be tested with six embryos per compound.

We chose 28-30 hpf as time point of analysis because, at this time, growth of the CaP axon beyond the HM can be assessed, without later extending secondary motor axons confounding the analysis (Myers et al., 1986). At this time point, in control embryos (*chodl+/+; mnx1:EGFP*), motor axons typically have all grown beyond the HM, whearas in chodl mutants CaP motor axons are mostly stalled at the HM (Opris¸oreanu et al., 2019; Zhong et al., 2012). Hence, growth of axons beyond the HM in the *chodl* mutant provides a robust read-out of any potential rescue activity of compounds (Fig. 1A,B).

**Fig. 2.**
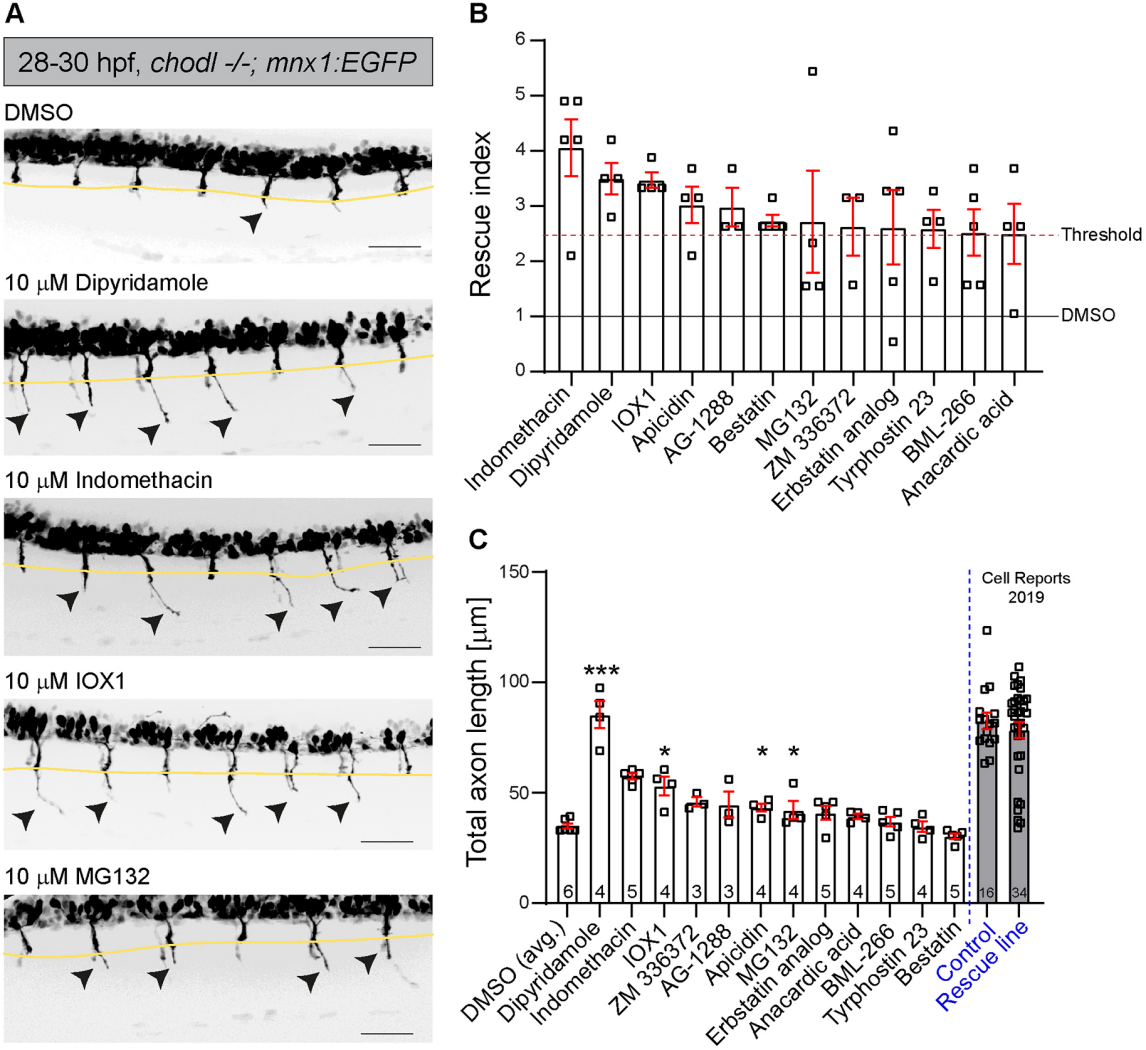
**Several compounds significantly increase the number of axons that grow beyond the HM.** (A) Representative VAST images of 28-29 hpf drug-treated *chodl* mutant embryos. Arrowheads indicate CaP motor axons beyond the HM (yellow line). Scale bars: 50 µm. (B) The 12 compound hits with the highest rescue index, presented according to the percentage of motor axons that crossed the HM in comparison to that of internal control (DMSO-*chodl* mutant). Compounds were tested at 10 µM. Each data point represents one animal. Error bars represent means±s.e.m. (C) The 12 compound hits are ranked according to the total length of CaP motor axons after compound application. The average length of CaP motor axons in DMSO-*chodl* mutant is 35.06 µm. Statistical tests were performed, comparing the drug treatment with its own DMSO-*chodl* mutant control (dipyridamole, Kruskal–Wallis test with Dunn's multiple comparison test ***P=0.0009, statistical power=0.999; IOX1, Mann–Whitney test *P=0.0159, statistical power=0.9750; MG132, Mann–Whitney test *P=0.0242, statistical power=0.9620; apicidin, Kruskal–Wallis test with Dunn's multiple comparison test *P=0.0306, statistical power=0.9564). Grey bars represent previously published data (Opris¸oreanu et al., 2019), and are used to estimate the effect of various compounds on the length of CaP motor axons compared to that in wild-type embryos (Control) and *chodl* mutant embryos, in which the phenotype was rescued by stable overexpression of *chodl* in motor neurons (Rescue line). Each data point represents one animal, *n*-numbers are indicated in each bar. Error bars represent means±s.e.m.

In the initial screening step, we scored the number of CaP motor axons that had passed the HM (eight axons from one side in segments 7-14). We normalised the number of axons grown beyond the HM in the compound-treated groups to that seen in the DMSO-*chodl* mutant group (internal control), thus, obtaining a rescue index. Typically, one or two axons had grown beyond the HM in controls. We set a rescue index of 2.5 as a threshold to identify ‘hits’. That would be reached, for example, if five out of eight axons grew beyond the HM compared to two out of eight in controls. We obtained 12 out of 982 compounds that passed this threshold: indomethacin, dipyridamole, IOX1, apicidin, AG-1288, bestatin hydrochloride, MG132, ZM 336372, the erbstatin analog methyl 2,5-dihydroxycinnamate, tyrphostin 23, BML-266 and anacardic acid (Fig. 2A,B, Fig. S1A,B). None of the 12 compounds induced visible changes in embryo development, i.e. no alterations regarding the gross morphology of embryos and the dorso-ventral extent of the trunk within the region where motor axon length was analysed (Fig. S1C,D).

Overall, out of all 982 small molecules screened, 47 induced death, 22 delayed embryo development and 13 led to severely malformed embryos. These drugs, amounting to 8.3% of the total compounds tested, were considered toxic and excluded from the analysis.

Although hit compounds rescued the axonal phenotype in the *chodl* mutant, the total length of these CaP axons varied between compounds – an observation not captured by the rescue index. Thus, we introduced a measurable parameter to compare the magnitudes of rescue effect of the compounds for our 12 hits. We measured the total length of the CaP motor axon (from the spinal exit point to the tip of the axon) in drug-treated embryos, comparing it to the DMSO control of the same data set. Out of the 12 compounds, four induced a statistically significant increase in the total axon length. Ranked according to their *P*-values, these were: (1) dipyridamole, a non-selective phosphodiesterase and adenosine uptake inhibitor (Ciacciarelli et al., 2015; Gamboa et al., 2005); (2) IOX1, a broad-spectrum inhibitor of 2OG oxygenases, including histone demethylases (Hopkinson et al., 2013); (3) the proteasome inhibitor MG132 (Inoue et al., 2004; Momose et al., 2002; Saito et al., 1992; Song et al., 2009) and (4) the histone deacetylase inhibitor apicidin (Darkin-Rattray et al., 1996) (Fig. 2C, Table 1). These ranks correspond to hit ranks 2, 3, 4 and 7 of the initial scoring (Fig. 2B). Overall, these data show reasonable similarity between score and measurement, and validate the quick scoring of axon length as a screening tool. Therefore, we decided to focus our further analysis on the top three compounds with significant effects on axon length, i.e. dipyridamole, IOX1 and MG132.

**Table 1.**
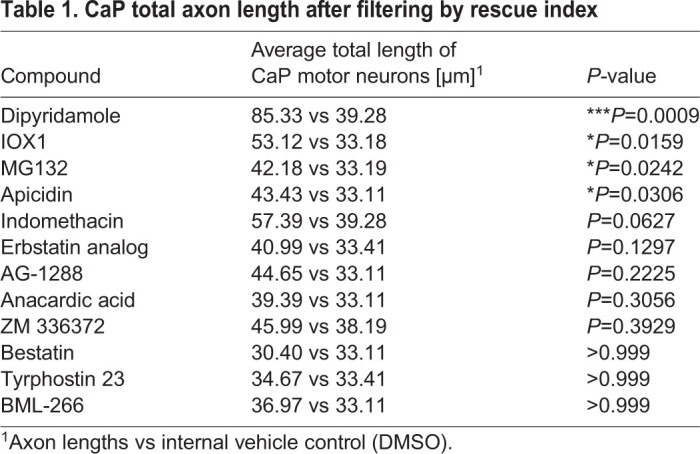
CaP total axon length after filtering by rescue index

### Validation of the rescue activity for the top hits

**Fig. 3.**
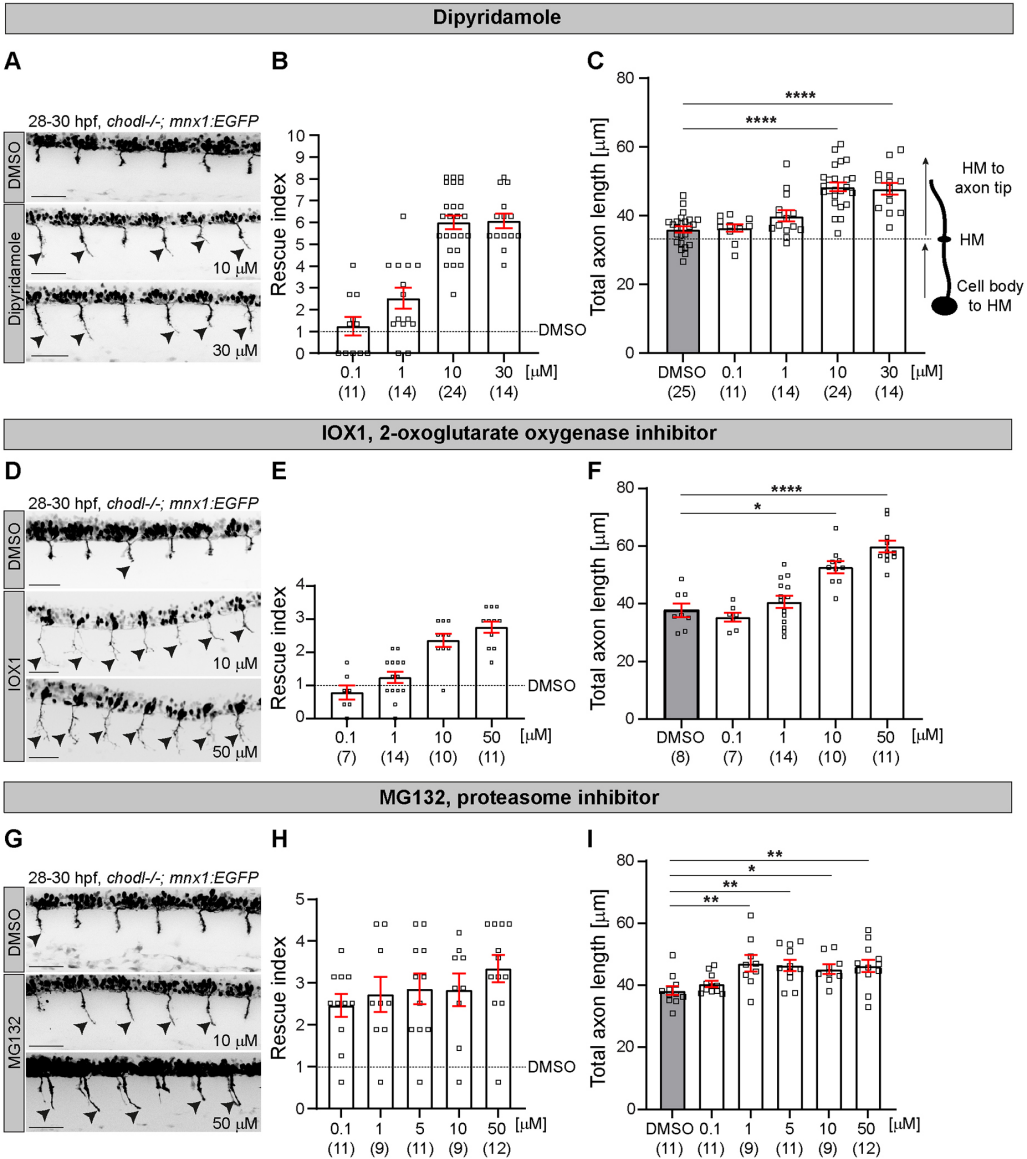
**Rescue of the axonal phenotype of the *chodl*−/− mutant at different concentrations of top drug hits.** (A,D,G) Representative images of the lateral view of *chodl* mutants treated with DMSO (control), dipyridamole (10 μm or 30 μm; A), IOX1 (10 μm or 50 μm; D) or MG132 (10 μm or 50 μm; G). Arrowheads indicate CaP motor axons beyond the HM. Scale bars: 50 µm. (B,E,H) Plotted is the growth of CaP motor axons beyond the HM in response to different concentrations of dipyridamole (B), IOX1 (E) or MG132 (H), as rescue index over drug concentration. Treatment with IOX1 shows a dose-dependent rescue of the axonal phenotype in *chodl* mutants. (C,F,I) Quantification of the total CaP axon length after treatment with dipyridamole (C), IOX1 (F) or MG132 (I) at different concentrations compared with the total length of Cap axons treated with DMSO (control DMSO-*chodl* mutant). Treatment with dipyridamole (C), one-way ANOVA ****P<0.0001 with Dunnett's multiple comparison test, ****P<0.0001, statistical power=1.0000. Treatment with IOX1 (F), Kruskal–Wallis test ****P<0.0001 with Dunn's multiple comparison test ****P<0.0001, *P=0.0177, statistical power=0.9940. Treatment with MG132 (I), one-way ANOVA **P=0.0022 with Dunnett's multiple comparison test: DMSO vs 1 µM **P=0.0060, DMSO vs 5 µM **P=0.0078, DMSO vs 10 µM *P=0.0422, DMSO vs 50 µM **P=0.0077, statistical power=0.9473). Each data point represents one animal and *n* numbers are indicated in parenthesis. Error bars represent means±s.e.m.

To exclude the possibility of a false-positive result, we determined the concentration dependence of the rescue activity in *chodl* mutants for dipyridamole, IOX1 and MG132 at concentrations between 0.1 µM and 30-50 µM. Indeed, dipyridamole rescued the axonal phenotype of the *chodl* mutant (Fig. 3A,B) and increased the average CaP axon length to 135% (10 µM) and 133% (30 µM) of the DMSO-treated embryos in a concentration-dependent manner (Fig. 3C).

Treatment with IOX1 also increased the average axon length to 139% (10 µM) and 158% (50 µM) compared with the DMSO-treated group in a concentration-dependent manner (Fig. 3D-F). Similarly, MG132 showed rescuing activity from 1 µM, which was, however, similar over a wide range of concentration (Fig. 3G,H). The average axon length increased starting with 1 µM MG132 compared with the DMSO-treated group (123% for 1 µM, 121% for 5 µM, 118% for 10 µM, and 121% for 50 µM) (Fig. 3I).

Growth of the main ventrally directed axon is the principal read-out of rescue for the *chodl* mutant. In wild-type animals, ventral growth of the CaP axon is the main mode of axon extension at the stages analysed. However, at the time point analysed (28-30 hpf), first side branches also develop. Therefore, we decided to assess whether IOX1 induces side branches. *chodl* mutants not treated or treated with IOX1 at concentrations up to 10 µM had fewer branches (>8 µm long) compared with identically treated wild-type embryos, probably due to the more immature state of the motor axon in the mutant. However, after 50 µM IOX1 treatment, the increase in branching matched that of wild-type control (Fig. S2A,B). In wild-type embryos, IOX1 treatment also trended towards a slight increase in axon branching compared to DMSO-treated (control) wild-type embryos. IOX1, therefore, seems to enhance the maturation of axons, a process during which increased branching occurs naturally. Alternatively, the drug might induce ectopic branching in addition to promoting longitudinal axonal growth as seen in the mutant. As we cannot differentiate between these two possibilities, we did not pursue branching phenotypes in *chodl* mutants.

Overall, our concentration-response experiments showed concentration dependence for dipyridamole, IOX1 and MG132, confirming the results of the first screening.

### Presynaptic defects in *chodl* mutants are rescued by drug treatment

**Fig. 4.**
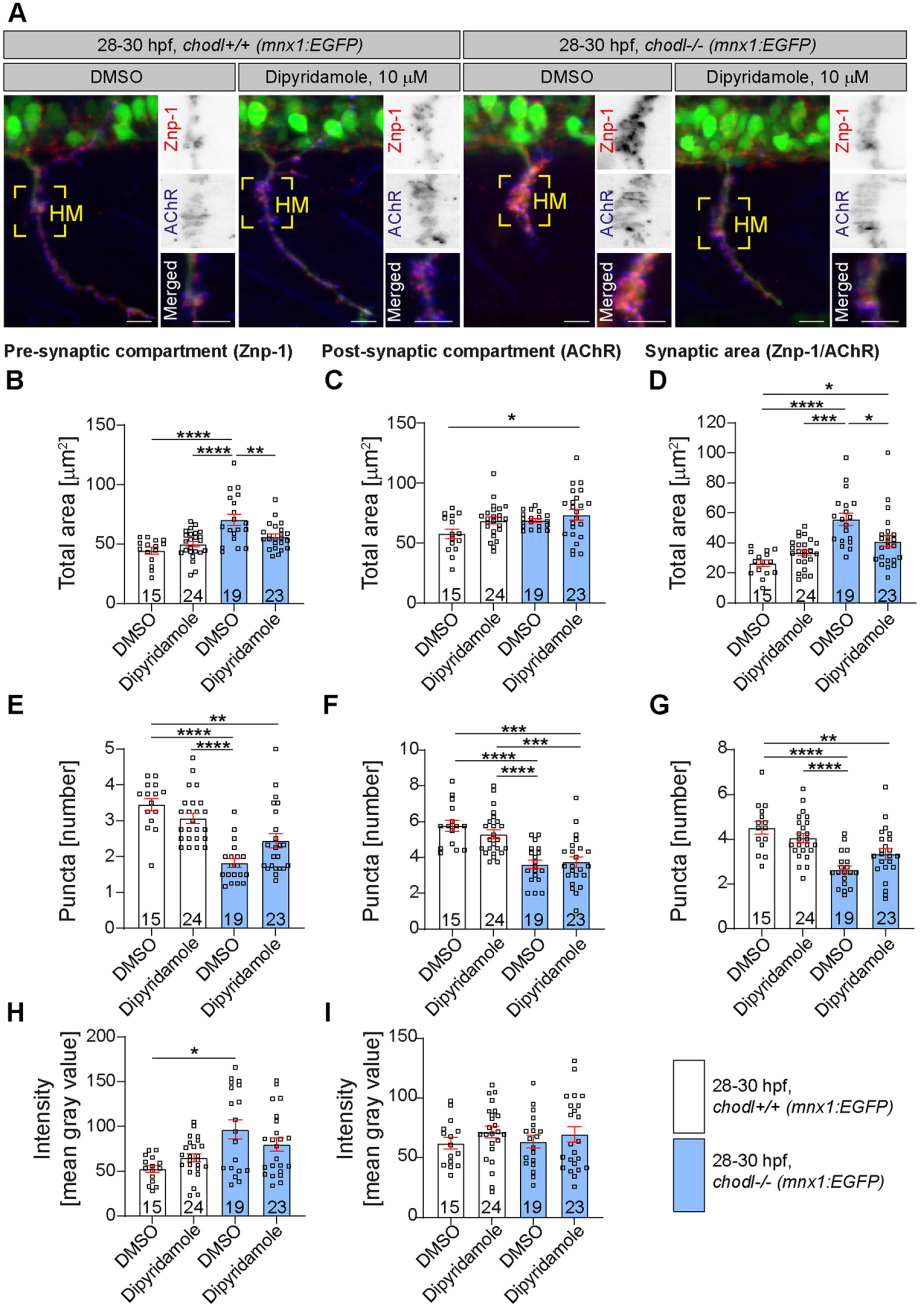
**Dipyridamole rescues presynaptic defects in the *chodl*−/−** **mutant.** (A) Representative images of motor axons in control *chodl*+/+ (*mnx1:EGFP*) and *chodl* mutant fish (*chodl−/−; mnx1:EGFP*) after control (DMSO) or drug (dipyridamole, 10 µM) treatment. Motor axons are labelled in green, the presynaptic compartment was labelled for the synaptic marker synaptotagmin-2 (red, using Znp-1) and the postsynaptic compartment by antibodies against AChR (blue). Yellow squares indicate the horizontal myoseptum (HM). Scale bars: 10 µm. (B-D) Quantification of the presynaptic (B), postsynaptic (C) and total (D) area in zebrafish embryos as described in A. (B) The presynaptic compartment is enlarged in *chodl* mutants but 10 µM dipyridamole rescues this phenotype (one-way ANOVA ****P<0.0001 with Tukey's multiple comparison test: DMSO-control vs DMSO-*chodl* mutant ****P<0.0001, dipyridamole-control vs DMSO-*chodl* mutant ****P<0.0001, DMSO-*chodl* mutant vs dipyridamole-*chodl* mutant **P=0.0090, statistical power=0.9999). (C) Dipyridamole induces an increase in the total postsynaptic area in *chodl* mutants (one-way ANOVA *P=0.0269 with Tukey's multiple comparison test *P=0.0148, statistical power=0.7222). (D) The enlarged total synaptic area in *chodl* mutants is not fully rescued by dipyridamole (Kruskal–Wallis test ****P<0.0001 with Dunn's multiple comparison test: DMSO-control vs DMSO-*chodl* mutant ****P<0.0001, DMSO-control vs dipyridamole-*chodl* mutant *P=0.0194, dipyridamole-control vs DMSO-*chodl* mutant ***P=0.0004, DMSO-*chodl* mutant vs dipyridamole-*chodl* mutant *P=0.0382, statistical power=0.9999). (E-G) Quantification of the presynaptic (E), postsynaptic (F) and total (G) number of discernible puncta in zebrafish embryos as described in A. (E) The reduced number of presynaptic discernible puncta in *chodl* mutants is not fully rescued by dipyridamole (Kruskal–Wallis test ****P<0.0001 with Dunn's multiple comparison test: DMSO-control vs DMSO-*chodl* mutant ****P<0.0001, DMSO-control vs dipyridamole-*chodl* mutant **P=0.0034, dipyridamole-control vs DMSO-*chodl* mutant ****P<0.0001, statistical power=0.9999). (F) The reduced number of postsynaptic discernible puncta in *chodl* mutants is not rescued by dipyridamole (one-way ANOVA ****P<0.0001 with Tukey's multiple comparison test: DMSO-control vs DMSO-*chodl* mutant ****P<0.0001, DMSO-control vs dipyridamole-*chodl* mutant ****P<0.0001, dipyridamole-control vs DMSO-*chodl* mutant ***P=0.0002, dipyridamole-control vs dipyridamole-*chodl* mutant ***P=0.003, statistical power=0.9999). (G) The total number of discernible puncta in *chodl* mutants is not rescued by dipyridamole (one-way ANOVA ****P<0.0001 with Tukey's multiple comparison test: DMSO-control vs DMSO-*chodl* mutant ****P<0.0001, DMSO-control vs dipyridamole-*chodl* mutant **P=0.0039, dipyridamole-control vs DMSO-*chodl* mutant ****P<0.0001, statistical power=0.9995). (H,I) Labelling intensity of pre- (H) and postsynaptic (I) area in zebrafish embryos as described in A. (H) The increased presynaptic labelling intensity in *chodl* mutant is not rescued by dipyridamole (Kruskal–Wallis test *P=0.0249 with Dunn's multiple comparison test: DMSO-control vs DMSO-*chodl* mutant *P=0.0213, statistical power=0.9517). (I) Application of dipyridamole does not change the mean labelling intensity of the postsynaptic compartment in different treatment groups. *chodl* mutants, blue bars; wild-type embryos, white bars; with drug application (dipyridamole) and without drug application (DMSO). Each data point represents one animal, *n*-numbers are indicated within each bar. Error bars represent means±s.e.m.

**Fig. 5.**
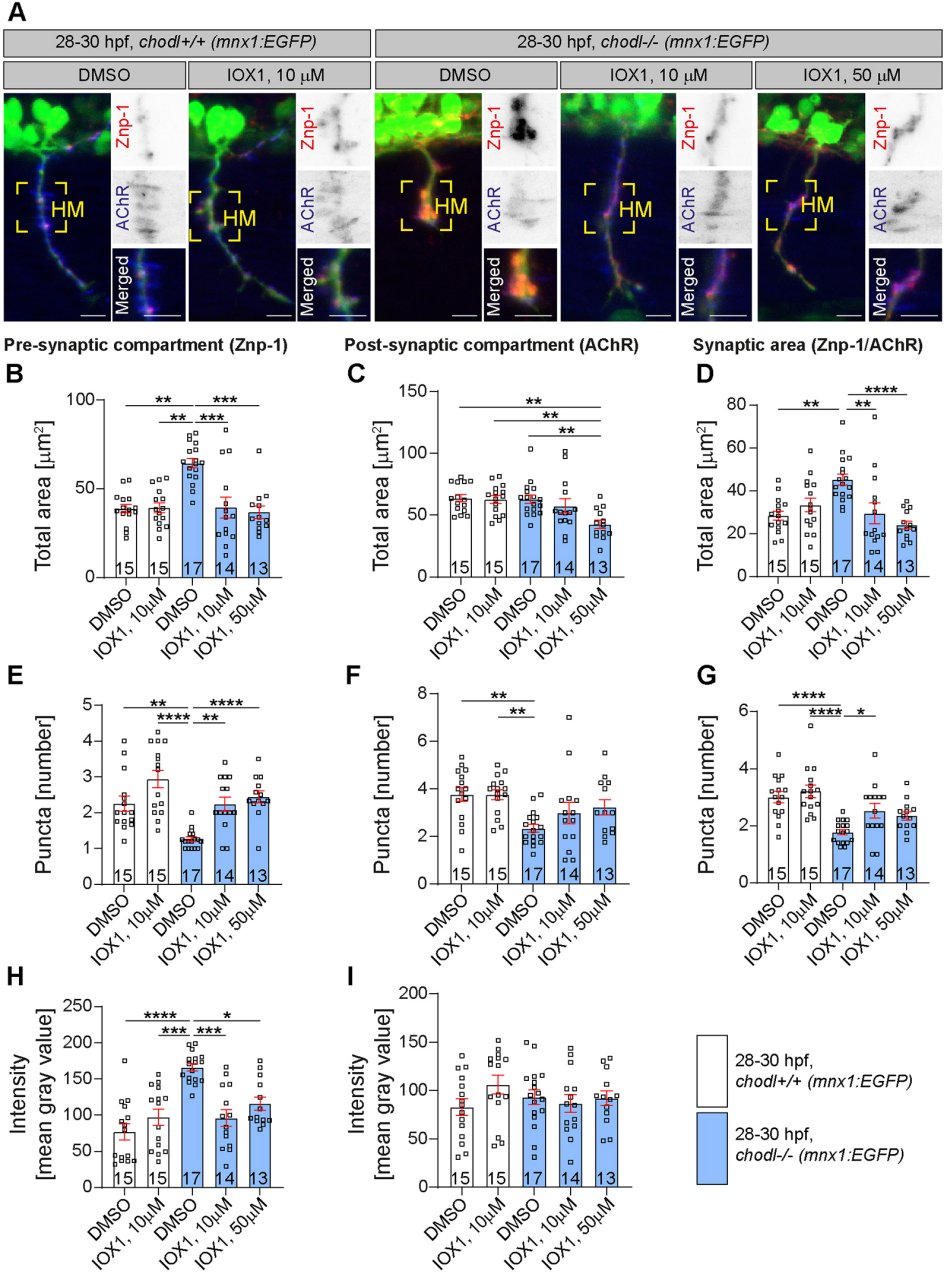
**Pre- and postsynaptic defects in *chodl*−/− mutants are rescued by IOX1.** (A) Representative images of motor axons in *chodl*+/+ (*mnx1:EGFP*) and *chodl* mutant fish (*chodl−/−; mnx1:EGFP*) after control (DMSO) or drug (IOX1, 10 µM and 10 µM or 50 µM, respectively) treatment. Motor axons are labelled in green, the presynaptic compartment was labelled for the synaptic marker synaptotagmin-2 (red, using Znp-1) and the postsynaptic compartment by antibodies against AChR (blue). Yellow squares indicate the horizontal myoseptum (HM). Scale bars: 10 µm. (B-D) Quantification of the presynaptic (B), postsynaptic (C) and total (D) area in zebrafish embryos as described in A. (D) The presynaptic compartment is enlarged in *chodl* mutants but application of IOX1 rescues this phenotype (Kruskal–Wallis test ****P<0.0001 with Dunn's multiple comparison test: DMSO-control vs DMSO-*chodl* mutant **P=0.0011, IOX1 10 µM-control vs DMSO-*chodl* mutant **P=0.0011, DMSO-*chodl* mutant vs IOX1 10 µM-*chodl* mutant ***P=0.0003, DMSO-*chodl* mutant vs IOX1 50 µM-*chodl* mutant ***P=0.0002, statistical power=0.9540). (C) Application of 50 µm of IOX1 induces a decrease in the postsynaptic total area in *chodl* mutants (Kruskal–Wallis test **P=0.0014 with Dunn's multiple comparison test: DMSO-control versus IOX1 50 µM-*chodl* mutant **P=0.0039, IOX1 10 µM-control vs IOX1 50 µM-*chodl* mutant **P=0.0072, DMSO-*chodl* mutant vs IOX1 50 µM-*chodl* mutant **P=0.0070, statistical power=0.9526). (D) The enlarged total synaptic area in *chodl* mutants is rescued by IOX1 (Kruskal–Wallis test ****P<0.0001 with Dunn's multiple comparison test: DMSO-control vs DMSO-*chodl* mutant **P=0.0052, DMSO-*chodl* mutant vs IOX1 10 µM-*chodl* mutant **P=0.0026, DMSO-*chodl* mutant vs IOX1 50 µM-*chodl* mutant ****P<0.0001, statistical power=0.9613). (E-G) Quantification of the presynaptic (E), postsynaptic (F) and total (G) number of discernible puncta in zebrafish embryos as described in A. (E) The reduction in the number of presynaptic discernible puncta in *chodl* mutants is rescued by application of IOX1 (Kruskal–Wallis test ****P<0.0001 with Dunn's multiple comparison test: DMSO-control vs DMSO-*chodl* mutant **P=0.0059, IOX1 10 µM-control vs DMSO-*chodl* mutant ****P<0.0001, DMSO-*chodl* mutant vs IOX1 10 µM-*chodl* mutant **P=0.0058, DMSO-*chod*l mutant vs IOX1 50 µM-*chodl* mutant ****P=0.0002, statistical power=0.9618). (F) IOX1 does not rescue the reduced number of discernible puncta for the postsynaptic compartment in *chodl* mutants (one-way ANOVA **P=0.0030 with Tukey's multiple comparison test: DMSO-control vs DMSO-*chodl* mutant **P=0.0059, IOX1 10 µM-control vs DMSO-*chodl* mutant **P=0.0064, statistical power=0.9560). (G) The total number of discernible puncta in *chodl* mutants is rescued by application of IOX1 (one-way ANOVA ****P<0.0001 with Tukey's multiple comparison test: DMSO-control vs DMSO-*chodl* mutant ****P<0.0001, IOX1 10 µM-control vs DMSO-*chodl* mutant ****P<0.0001, DMSO-*chodl* mutant vs IOX1 10 µM-*chodl* mutant *P=0.0359, statistical power=0.9528). (H,I) Labelling intensity of pre- (H) and postsynaptic (I) area in zebrafish embryos as described in A. (H) The increased presynaptic labelling intensity in *chodl* mutant is rescued by IOX1 application (Kruskal–Wallis test ****P<0.0001 with Dunn's multiple comparison test: DMSO-control vs DMSO-*chodl* mutant ****P<0.0001, IOX1 10 µM-control vs DMSO-chodl mutant ***P=0.0004, DMSO-chodl mutant vs IOX1 10 µM-chodl mutant ***P=0.0005, DMSO-chodl mutant vs IOX1 50 µM-chodl mutant *P=0.0359, statistical power=0.9999). (I) The mean intensity of the postsynaptic compartment is unchanged between different treatment groups. *chodl* mutants, blue bars; wild-type embryos, white bars; with drug application (IOX1) and without drug application (DMSO). Each data point represents one animal, *n*-numbers are indicated within each bar. Error bars represent means±s.e.m.

The premise of our screen was that rescued axon length reflects rescue of synapse morphology at the horizontal myoseptum synaptic site. Because treatment with either drug might affect axon growth independently of synapse formation at 28-30 hpf, we tested whether application of the top hit drugs dipyridamole or IOX1 also rescues the synapse morphology at the horizontal myoseptum synaptic site in the *chodl* mutant. We labelled synapses at the horizontal myoseptum synaptic site against the synaptic marker synaptotagmin-2 (by using Znp-1 antibody) and against the postsynaptic marker acetylcholine receptor (AChR) (Figs 4A and 5A). We then determined the size of pre- and postsynapses and their overlap. A second sensitive measure of synapse morphology is to count the number of discernible synaptic labelling areas (puncta). Finally, we determined labelling intensity of puncta, as described (Opris¸oreanu et al., 2019).

For *chodl* mutants, we have previously reported an increase in the presynaptic area, which is related to a decrease in the number of discernible puncta, and increased labelling intensity of synaptotagmin-2 (after using Znp-1 antibody). For the postsynapse, we have only observed minor changes in the number of puncta for the postsynapse – probably a secondary consequence of the cell-autonomous changes in the presynapse (Opris¸oreanu et al., 2019). Here, we analysed the potential rescue of these parameters through the use of our top hit drugs.

### Dipyridamole

For DMSO-treated controls, we found that, in accordance with previous observations (Opris¸oreanu et al., 2019), DMSO-*chodl* mutants showed a 58% larger presynaptic compartment (Fig. 4B) and a 111% larger total synaptic area compared to DMSO-treated wild-type controls (Fig. 4D). The puncta number was reduced in the mutant for presynapse, postsynapse and overlap (Fig. 4E-G). The labelling intensity of synaptotagmin-2 at the presynapse was increased by 83% in the DMSO-treated *chodl* mutant (Fig. 4H), whereas the mean intensity of AChR (postsynapse) was not affected (Fig. 4I).

In the presence of 10 µM dipyridamole, the size of the presynaptic compartment was fully rescued (Fig. 4B). However, the number of presynaptic puncta was not statistically significantly rescued by dipyridamole (Fig. 4E). The increased intensity of synaptotagmin-2 labelling was also not rescued by dipyridamole treatment (Fig. 4H). Treatment of control embryos (wild-type) with 10 µM dipyridamole did neither alter area nor the puncta number of the presynaptic compartment indicating that the drug was not toxic.

The postsynaptic area in *chodl* mutants did not differ significantly from wild-type animals and addition of dipyridamole to the mutants did not alter this parameter (Fig. 4C). However, the more-sensitive measure of puncta number showed a reduction of 37% in the mutant compared to DMSO-treated wild-type control that was not rescued by the drug (Fig. 4F). Lack of *chodl* or treatment of either embryo type with dipyridamole did not affect the labelling intensity of the postsynapse compartment (Fig. 4I).

In *chodl* mutants, the total synaptic area was rescued by dipyridamole, albeit not to control levels (Fig. 4D), and the number of synaptic puncta was not restored to control (wild-type) levels (Fig. 4G). These findings suggest that dipyridamole partially rescues the abnormally large synapses at the horizontal myoseptum synaptic site in *chodl* mutants and that this rescue is driven mainly by the reduction in the size of the presynapse.

### IOX1

The DMSO-treated *chodl* mutants showed similar phenotypes compared to wild type as to those described above for dipyridamole experiments. Application of 10 µM and 50 µM IOX1 completely rescued the size of the presynaptic area in the *chodl* mutant to wild-type control levels (Fig. 5B). The number of presynaptic discernible puncta was also restored after IOX1 treatment (Fig. 5E). Mean intensity of the synaptotagmin-2 labelling was rescued by both 10 µM and 50 µM IOX1 (Fig. 5H). Treatment of wild-type embryos with IOX1 had no effect on synapse size, indicating that the drug was not toxic to motor axon development (Fig. 5).

The total postsynaptic area, labelled by anti-AChR antibodies, was not altered in *chodl* mutants compared to wild-type controls, as previously observed (Opris¸oreanu et al., 2019). However, application of IOX1 slightly decreased the size of the postsynaptic area in the treated mutants at the higher concentration of 50 µM (32% with 50 µM IOX1 in comparison to DMSO-treated mutants; Fig. 5C). The number of postsynaptic discernible puncta, which was reduced in the mutant, was not rescued by IOX1 (Fig. 5F). The labelling intensity of the postsynaptic compartment was not affected by *chodl* mutation or by treatment with IOX1 (Fig. 5I).

The overlap between the pre- and postsynaptic areas that represents the synaptic area was rescued by both concentrations of IOX1 (Fig. 5D). The number of discernible puncta of synaptic overlap was also rescued by 10 µM IOX1 (Fig. 5G). Together, these findings indicate that IOX1 strongly rescues synapse morphology, driven by rescue of all presynaptic defects in the *chodl* mutants.

### Dipyridamole partially rescues the phenotype of the UBA1 model for SMA

We hypothesized that, if similar synapse-related mechanisms are affected, active compounds that can rescue the *chodl* mutant also rescue the phenotype of SMA models. To test this, we established the UBA1 model of SMA (Wishart et al., 2014), by using quercetin rescue as a control. In contrast to the *chodl* mutant, where the main phenotype is a reduced axon length, the UBA1 model displays a range of axonal abnormalities, including aberrant axonal branching, stalled axons or absent axon outgrowth. This necessitated a different evaluation system that scored axons as ‘normal’ or ‘abnormal’ according to established criteria (see Materials and Methods, Wishart et al., 2014). Indeed, inhibition of UBA1 by using the UBEI-41 inhibitor led to a 117% increase in the number of abnormal axons compared to DMSO-treated embryos (Fig. S3A,B). This phenotype was rescued – by using the rescue in response to 50 µM quercetin as a positive control – to levels that were not significantly different from controls anymore (Fig. S3A,B), indicating sensitive detection of rescue activity in the model.

**Fig. 6.**
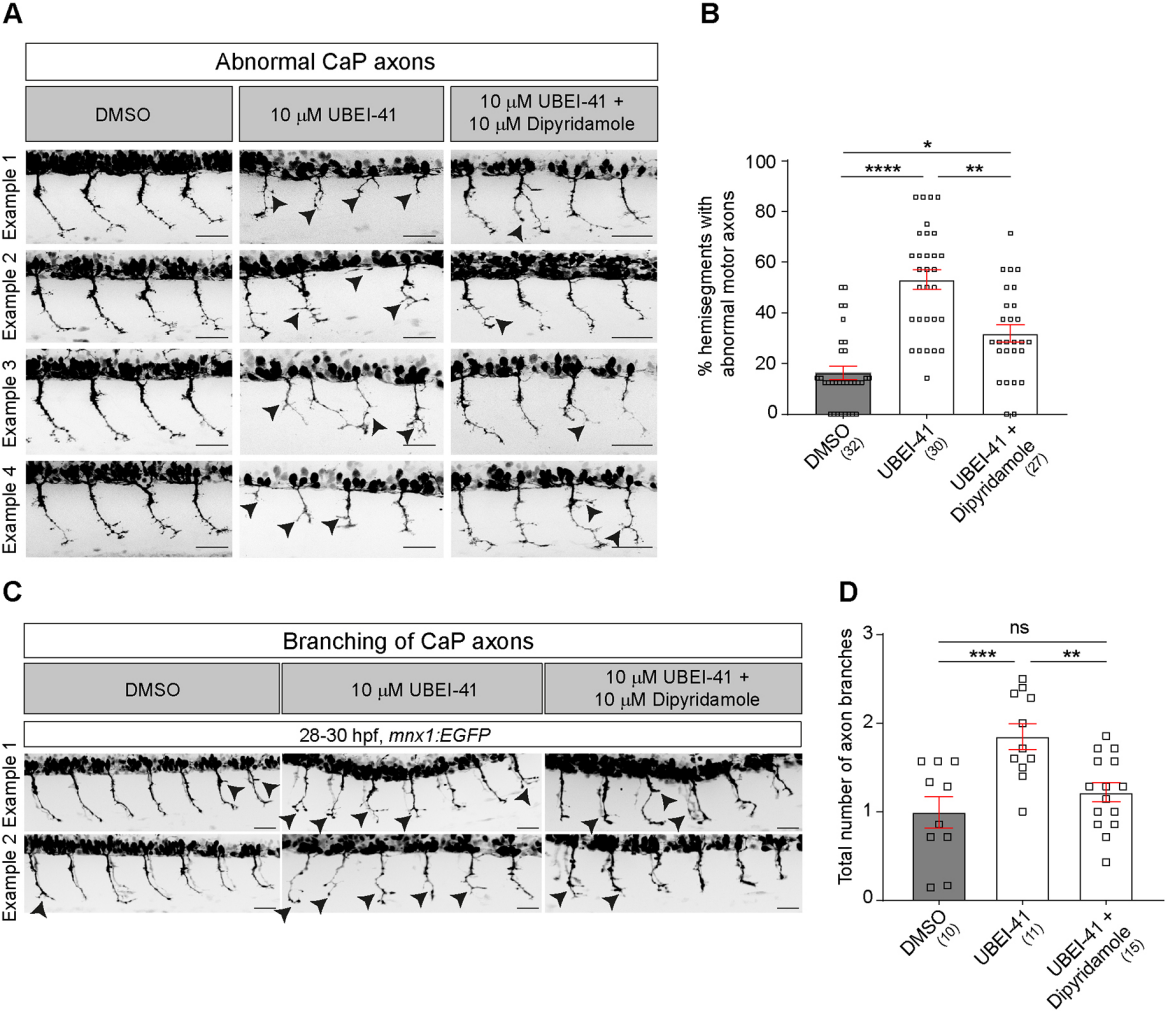
**Dipyridamole partially rescues abnormal axon morphology in the UBEI-41 model of SMA.** (A) Representative images of the lateral trunk of 28-30 hpf wild-type embryos. Abnormal motor neurons, i.e. short, missing or branched axons, are indicated by arrowheads. (B) The percentage of abnormal motor axons is decreased in embryos after combined treatment with UBEI-41 and dipyridamole (10 µM each) compared to those treated with UBEI-41 alone (Kruskal–Wallis test ****P<0.0001 with Dunn's multiple comparison test: ****P<0.0001, **P=0.0078 *P=0.0124, statistical power=0.999). (C) Representative images of 28-30 hpf wild-type embryos treated with DMSO, UBEI-41 or UBEI-41 and dipyridamole are shown. Abnormal axon branching is indicated by black arrowheads. Scale bars: 50 µm. (D) Quantification of axonal branching only. The number of axon branches is increased in embryos after treatment with dipyridamole (10 µM) compared to those treated with DMSO (control) (one-way ANOVA test ***P=0.0006 with Tukey's multiple comparison test: ***P=0.0007, **P=0.0062 statistical power=0.9618). All scale bars: 50 µm. Each data point represents one animal and *n* numbers are indicated in parenthesis. Error bars represent means±s.e.m.

Next, we tested whether the hit compounds rescue the axonal abnormalities associated with the UBA1 model. We found that 10 µM dipyridamole induced a 31% decrease in the percentage of motor axons with abnormal morphology in UBEI-41-treated fish compared to vehicle- and UBEI-41-treated controls (Fig. 6A,B). This represents a partial rescue of the phenotype because the proportion of abnormal axons was still higher than in vehicle-only treated embryos.

A sub-analysis of only aberrant branching showed that this phenotype was rescued to a level that was not statistically different from DMSO-treated controls anymore (Fig. 6C,D). Hence, dipyridamole has a strong rescuing activity of axonal phenotypes in the UBEI-41 model of SMA.

In contrast to the above compound, incubation of UBEI-41-treated zebrafish embryos with 10 µM IOX1 or MG132 exacerbated the abnormal axonal phenotype rather than rescuing it (Fig. S3).

To investigate the possibility of translating the dipyridamole findings from this SMA model to other MND models, we used the C9orf72 model of ALS, which shows shorter and abnormally branched motor axons (Ciura et al., 2013). Treatment of C9orf72 knockdown zebrafish with 10 µM dipyridamole did not rescue the phenotype of reduced axon length (Fig. S4). This indicates potential additional mechanisms in this model compared to the mechanisms causing the phenotypes observed in the *chodl* or UBA1 models.

**Fig. 7.**
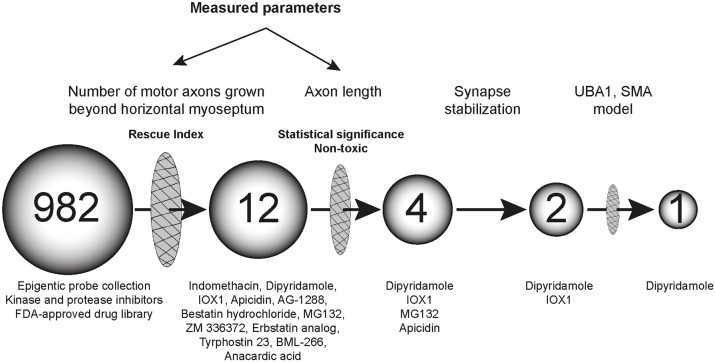
**Schematic representation of the results.** Out of 982 chemical compounds tested, four rescue the axonal phenotype of *chodl* mutants and one significantly improves axon morphology in the UBA1–SMA model.

In summary, by screening 982 compounds for synapse stabilisation in the zebrafish *chodl* mutant we found that dipyridamole partially rescued the axonal phenotype in a zebrafish SMA model (Fig. 7).

## DISCUSSION

We have successfully established an *in-vivo* phenotypic screening platform to find molecules that stabilise synapses at the horizontal myoseptum synaptic site in zebrafish. These molecules may be relevant for ameliorating synapse-related deficits in motor neuron diseases; indeed, we show that dipyridamole rescues defects in the *chodl* mutant and in a zebrafish SMA model.

We used the *chodl* mutant to detect chemical compounds that stabilize synapses in a large group of target-annotated small-molecule compounds and approved drugs. The main advantage of using this zebrafish mutant is its robust phenotype, with >75% stalled CaP motor axons at the HM. In zebrafish, the growth of motor axons depends on *en passant* neuromuscular synapse formation at the HM (Opris¸oreanu et al., 2019). We reasoned that compounds that stabilize synapses at this horizontal myoseptum synaptic site in the *chodl* mutant lead to an increase in axon length, and that this could be used as a robust and quick read-out for proper synapse formation. Indeed, for two compounds, dipyridamole and IOX1, we showed that these not only cause increased CaP axon length but that they successfully rescue presynaptic defects in *chodl* mutants at the horizontal myoseptum synaptic site. These observations indicate that our screen detects compounds that stabilize synapse morphology.

Our screening protocol is fast. For example, it takes ∼4 h to test 20 compounds on 144 embryos. Manual mounting and imaging would take 8 h (2 h mounting and 6 h confocal imaging). Screening is facilitated by establishing new experimental settings on the VAST BioImager for automatic loading, positioning and imaging of 28-30 hpf embryos. The VAST BioImager platform has been previously shown to work very well with 48 hpf (Pardo-Martin et al., 2010), 72 hpf (Ansar et al., 2019) and 96 hpf larvae (Early et al., 2018), but the fragility and large yolk sac mostly prevented use on younger embryos. Another issue with younger embryos is their sparse pigmentation, which impairs recognition of the embryo as it passes through the detection light-gate and, thus, disrupts the ability of the VAST BioImager to match the correct sample orientation for imaging. Since many biological processes occur during earlier stages, we have significantly extended the capacity for automated zebrafish imaging.

After screening 982 compounds, we found 12 compounds that passed the rescue index threshold; of these, four compounds resulted in significantly longer CaP axons. This gives us a hit rate of 1.22% (12/982) and 0.40% (4/982) for first- and second-stage screening. These hit rates are comparable with other library screens in zebrafish, e.g. 1.25% (4/320) in a screen for compounds that alleviate seizure (Baraban et al., 2013), 0.85% (17/2000) in a screen for inhibitors of fin regeneration (Mathew et al., 2007), and 0.4% (4/1000) in a screen for compounds to treat Dravet syndrome (Dinday and Baraban, 2015). However, it is difficult to determine the rate of false-positive and false-negative results, as we might have missed compounds that stabilise synapses but inhibit axon growth – since axon length is our read-out. Following the same logic, compounds and drugs that only promote axon growth through mechanisms like cytoskeleton stabilization might override the need for synapse stabilisation and produce false-positive results. However, since two cytoskeleton-stabilizing FDA drugs, i.e. paclitaxel (also known as taxol) and docetaxel (also known as taxotere) that may force axon growth (Hellal et al., 2011) independently of synapse formation, did not rescue the *chodl* axon growth, this speaks against this possibility. To mitigate the risk of false-negative and false-positive results in a chemical screen, one could carry out screens at different concentrations. As a ‘compromise concentration’ that is within the range of activity for many small-molecule compounds in zebrafish and typically used in zebrafish *in vivo* screens (McGown et al., 2016; Yang et al., 2018), we decided on a concentration of 10 µM. Our titration for IOX1 confirms that 10 µM had almost maximal effect. Using more than one concentration would have decreased the screening capacity from 20 compounds to ten (two concentrations) or seven (three concentrations) compounds per imaging session. Our approach, thus, represents a compromise at the expense of a potential increase of false negative results.

In line with the notion that *chodl* is a key factor downstream of the SMN pathway and that synapse loss precedes neuronal death in SMA (Bäumer et al., 2009; Zhang et al., 2008), we reasoned that compounds that rescue synaptic defects in *chodl* mutants might also ameliorate defects in SMA models. Indeed, dipyridamole partially rescued the abnormal axon phenotype in the UBA1 model (Wishart et al., 2014). Dipyridamole blocks the uptake of adenosine and inhibits phosphodiesterases (PDEs) (Ciacciarelli et al., 2015; Gamboa et al., 2005), both processes that could be related to stability and function of the neuromuscular synapse. Dipyridamole inhibits adenosine uptake at the neuromuscular synapse in frogs (Ribeiro and Sebastião, 1987) and synaptic adenosine autoreceptors play a role in developmental synapse elimination in mammals (Tomàs et al., 2018). Cyclic nucleotides that are regulated by PDEs are involved in synaptic refinement of the neuromuscular synapse in *Drosophila* (Vonhoff and Keshishian, 2017). In addition, dipyridamole has been shown to be neuroprotective in cultured CNS neurons by acting as an anti-oxidant (Farinelli et al., 1998) and was identified as top hit in a screen looking for compounds with neuroprotective efficacy against α-synuclein-induced toxicity (Höllerhage et al., 2017). Dipyridamole also protects cultured motor neurons against chronic excitotoxicity by elevating the intracellular level of cGMP though inhibiting PDE5 (Nakamizo et al., 2003). Hence, there is accumulating evidence for neuroprotection by dipyridamole, which may include synapse protection.

Interestingly, neither IOX1 nor MG132 ameliorated the axonal defects in the UBA1 model but, instead, exacerbated these defects. A previous study on human iPSC-derived motor neurons has shown that MG132 or UBEI-41 treatment significantly decreased their viability (Bax et al., 2019). Hence, in our experiments, simultaneous application of MG132 and UBEI-41 might have resulted in synergistic motor axon toxicity. A similar effect might have occurred in IOX1-treated UBA1 embryos, although IOX1 by itself did not induce any abnormal branching or synaptic defects in treated wild-type embryos.

Overall, our study shows the usefulness of the zebrafish model to perform automated *in vivo* screening for drugs and compounds that stabilise the developing neuromuscular synapse. Moreover, by extending the capacity of automatic imaging to earlier developmental stages (28-30 hpf), the VAST system could be used in future studies that have primary motor axon growth as read-out. A wide range of zebrafish models could be suitable for our automated system, such as the established gene-disruption models of C9orf72, TDP43 and SOD1 (Lissouba et al., 2018; Robinson et al., 2019) for ALS, and the neurexin mutants for SMA (Koh et al., 2020). Furthermore, motor axon aberrations in *neuroserpin* mutants could be used to screen for neurodegenerative disorders (Han et al., 2021). Finally, the VAST BioImager platform can be used for phenotypic screening across a broader range of non-neural *in vivo* models and biological endpoints at 28-30 hpf, where cell types of interest are transgenically labelled (Chang and Weinstein, 2007). Compound hits from such phenotypic screens can be rapidly translated to other models of neurodegenerative and other diseases, to support further validation and mechanistic analysis.

## MATERIALS AND METHODS

### Animals

Zebrafish lines used in this work were the wik wild-type strain (Rauch, et al., 1997), the Tg(*mnx1*:EGFP) motor neuron reporter line (abbreviated as *mnx1*:GFP; Flanagan-Steet et al., 2005), and the *chodl* mutant line on the background of the *mnx1:*GFP reporter line (*chodl−/−; mnx1:*EGFP) (Opris¸oreanu et al., 2019). All fish were kept and bred in our laboratory fish facility according to standard procedures (Westerfield, 2007). All experimental procedures were performed on fish younger than 5 dpf. The *chodl* mutants (F3 homozygotes) used for drug screening were maintained in four or five tanks (3-litre tanks, with two female and two male fish per tank). Mating of *chodl* mutants was set up on a rotating basis, with a total of four pairs mating per week. Breeding protocols were approved by the British Home Office (project licence no. 70/8805).

### Compound incubation protocol for screening

At 6 hpf, zebrafish embryos were mechanically dechorionated using insect pins. Embryos were arrayed in a 24-well plate, with six fish per well, containing 10 µM of chemical compounds in embryo medium (E3). Embryos were allowed to develop at standard temperature (28.5°C) until imaging at 28 hpf. Chemical compounds used in the screen were from Epigenetic Probes Collection (40 compounds, Structural Genomic Consortium, UK), Kinase, Protease and Epigenetic inhibitors (176 compounds, Enzo SCREEN-WELL libraries, UK) and the Screen-well FDA approved drug library V2 version 1.0 (786 compounds, Enzo, UK). The stock concentration was at 10 mM in DMSO. For controls and re-testing the different hits, compounds were purchased from different suppliers and solubilized according to the manufacturer's instructions (stock concentration of 50 or 100 µM in DMSO): IOX1 (Cayman Chemicals, UK, cat. no. 11572), MG132 (Calbiochem, UK, cat. no. 474790), dipyridamole (Sigma, cat. no. D9766), UBEI-41 (Sigma, cat. no. N2915), and quercetin (Cayman Chemicals, UK, cat. no. 10005169).

### Automated image capture

To provide sufficient space for embryos to develop unimpeded, animals were raised in 24-well plates, with six embryos per well. At 28 hpf, embryos were moved from the 24-well plate to a 96-well plate (with three fish per well) to fit the loading bay of the Large Particle (LP) Sampler (Union Biometrica, Holliston, MA). The DMSO-treated internal control embryos (chodl mutants) were differently positioned into 96-well plates, i.e. into the first two wells, four random wells in the middle of the plate, and the last two wells to control for development of the embryos during the time needed for image capture. From the 96-well plate, embryos were automatically loaded by the LP Sampler and delivered to the VAST BioImager platform (Union Biometrica, Holliston, MA). Drugs or compounds that induced death (opaque embryos) or delayed development were excluded from loading. Delayed development was assessed as an embryo having a short and curved tail (segmentation period 20-25 somites). Other excluded embryos were in the earlier segmentation period (14-18 somites). The VAST BioImager platform was mounted on an Axio Examiner D1 (Carl Zeiss Microscopy, Jena, Germany) microscope fitted with a high-speed CSU-X1 spinning disk confocal scanner (Yokogawa, Tokyo, Japan) and a high-speed piezo drive to generate rapid *z-*stacks for image capture (Early et al., 2018).

The VAST experimental setting had to be optimized for embryos at 28-30 hpf; VAST loading speed and pump pressure were reduced to minimize the damage caused by the hydraulic pump on 28-30 hpf embryos (90 arbitrary units). To compensate for the tendency of embryos to clump together in the glass capillary, flushing and unloading volumes were set to 160 µl unloading volume and maximum of 300 µl between flushes. To increase detection of embryos with weaker pigmentation, the light intensity for objects was set to 10% (minimum intensity drop). This caused occasional imaging errors, i.e. embryos were discarded as being wrongly positioned or not correctly matching the previously user-defined template embryo shape. To correct for the lack of template differences, the minimum similarity to the template was set to 0.15°.

Embryos were rotated in the imaging chamber (thin-walled capillary glass with an internal diameter of 600 µm, provided by Union Biometrica, Holliston, MA) for a lateral view and imaged using brightfield and GFP channels. The output CSV format files containing number of fish, location of well, and orientation and time of acquisition, were processed with a Zen software (Zeiss) macro to generate the maximum intensity projections (MIPs) for each stack (Early et al., 2018). The whole embryo was captured in three parts (head, body, tail). For one embryo 12 MIPs were generated (four MIPs per body part, with two MIPs for the EGFP channel and two for the BF channel). MIPs were stitched together to obtain two tiff files of the entire fish (brightfield and GFP), using a previously published ImageJ macro (Early et al., 2018). These files were used to score the percentage of the motor axons that had passed the HM and to measure total axon length [NeuronJ plugin for ImageJ software (Meijering et al., 2004)]. For the entire experiment and analysis, the observer was blinded to the treatment.

### Rescue index, total axon length analysis and motor axon morphology scoring

For the axonal scoring, eight axons from the hemisegments closest to the objective, i.e. somites 7-14, were scored per embryo as either stalled at or dorsal to the HM, or grown beyond horizontal myoseptum (HM+). Next, the number of motor axons grown beyond the HM was normalized to the internal control (DMSO-treated group, see Results) to obtain the rescue index.

To measure axon length, axons were manually traced from their spinal exit point to their tips; total axon length was automatically determined using NeuroJ plugin in ImageJ. Despite working with projections, in the vast majority of cases we were able to trace and discern those axons closer to the objective, guided by the offset of their ventral root exit points and their increased brightness.

To quantify branching of *chodl* mutant axons at 28 hpf, maximum intensity projections of the trunk were generated. Branching for eight axons from somites 7-14 was quantified for each embryo. The number of branches were counted by tracing lines from the spinal cord ventrally, at a distance of 8 μm anterior and posterior from the main axon. Branches that crossed each line were counted. This counted the number of branches, but disregarded small filopodia or very minor branches coming from the axon. Branches on both sides of the axon were summated and averaged for each embryo.

Scoring of abnormal motor axon morphology in the UBA1 zebrafish SMA model was done according to Wishart et al. (2014). Briefly, motor axons with branches that are not normally observed at this developmental stage and truncated or missing axons were categorized as abnormal. Between six and seven axons from one hemisegment (from somite 7) per embryo were scored. During all analyses the observer was blinded to the treatments.

### Zebrafish whole-mount immunohistochemistry and synapse quantification

Using a previously published protocol (Opris¸oreanu et al., 2019), the 28-30 hpf zebrafish embryos were dechorionated, fixed and stained for pre- and postsynaptic compartments using primary antibodies mouse Znp-1 (1:100) against synaptotagmin-2 and rat mAb35 (1:200) against acetylcholine receptor (AChR) nicotinic alpha 1 subunit (both DSHB). Secondary antibodies were Alexa Fluor 594 donkey anti-mouse with pre-adsorption against rat protein and Alexa Fluor 647 anti-rat with pre-adsorption against mouse (both 1:400, Jackson ImmunoResearch). Synaptic puncta quantification was performed in ImageJ using a previously established protocol (Opris¸oreanu et al., 2019). Briefly, a square 400 µm^2^ region of interest was drawn around the HM. For each channel, the background was subtracted and a 30% threshold was applied to generate a binary image. Then, the Analyse Puncta function in ImageJ was used to generate outline masks to identify synaptic puncta. Puncta number and the area covered by puncta was then determined for four to five hemisegments per embryo and values were averaged for each embryo. The entire analysis was performed without knowledge of experimental condition.

### Analysis of total axon length and scoring of motor axon morphology in the C9orf72 model

Fertilised eggs from *mnx1:EGFP* zebrafish were microinjected at the single-cell stage with an antisense oligonucleotide targeting the ATG sequence of endogenous *C9orf72* (5′-ATTGTGGAGGACAGGCTGAAGACAT-3′; 1 nl of 0.02 mM; Gene Tools) as previously described (Ciura et al., 2013). At 6 h post fertilisation, eggs were moved to fresh water, and half of them treated with 10 µM dipyridamole. At 30 h post fertilisation, embryos were collected and dechorionated before being fixed in 4% PFA for 3 h at room temperature, washed with PBS-T then stored in 70% glycerol at 4°C overnight. Embryos were deyolked and whole-mounted for imaging on a Zeiss AxioImager Z1 microscope with ApoTome.2. Six pairs of motor axons were imaged per fish as *z*-stacks, beginning with the first pair after the yolk sac, over the yolk extension. Then, *z*-stacks were converted to maximum intensity projections for each side of the fish. Images were blinded for analysis. Axon length was measured using the Simple Neurite Tracer plug-in for ImageJ, and the average length per fish was plotted and analysed using Kruskal–Wallis with Dunn's multiple comparison test. For branching phenotype analysis, each axon was scored, i.e. 3=healthy neuron, 2=mild phenotype, 1=moderate phenotype, 0=severe phenotype, as previously described (Boyd et al., 2017). The percentage of healthy, mild, moderate and severe axons per fish were plotted and analysed using a two-way ANOVA with Tukey's multiple comparisons test.

### Microscopy and image acquisition

For whole-mount immunohistochemistry, zebrafish embryos were deyolked and mounted onto glass slides with 70% glycerol. For synapse quantifications, images were acquired using a Zeiss LSM880 with Airyscan, Axio examiner confocal microscope with Plan-Apochromat 20×/0.8 air objective.

To image abnormal motor axons in response to various combinations of drug treatment, a Zeiss AxioImager Z1, with ApoTome.2 upright microscope was used.

### Statistical analysis

Quantitative data were assessed for normality and analysed with parametric (one-way and two-way ANOVA with Tukey's or Dunnett's multiple comparison test) or non-parametric (Kruskal–Wallis test with Dunn's multiple comparison test, Mann–Whitney *U*-test) tests, as appropriate. Graphs were created using GraphPad Prism 9. Statistical power calculation was done using the G*Power 3.1, the aim for most experiments being >0.8. All figures were created using Adobe Photoshop CC and Adobe Illustrator CC.

## Supplementary Material

Supplementary information
