# Supplementary material for: Automated in vivo drug screen in zebrafish identifies synapse-stabilising drugs with relevance to spinal muscular atrophy
Source: Dis Model Mech. 2021 Apr 26;14(4):dmm047761. doi: 10.1242/dmm.047761 (PMC8106959; doi:10.1242/dmm.047761)
Supplement: Supplementary information [file dmm-14-047761-s1.pdf]

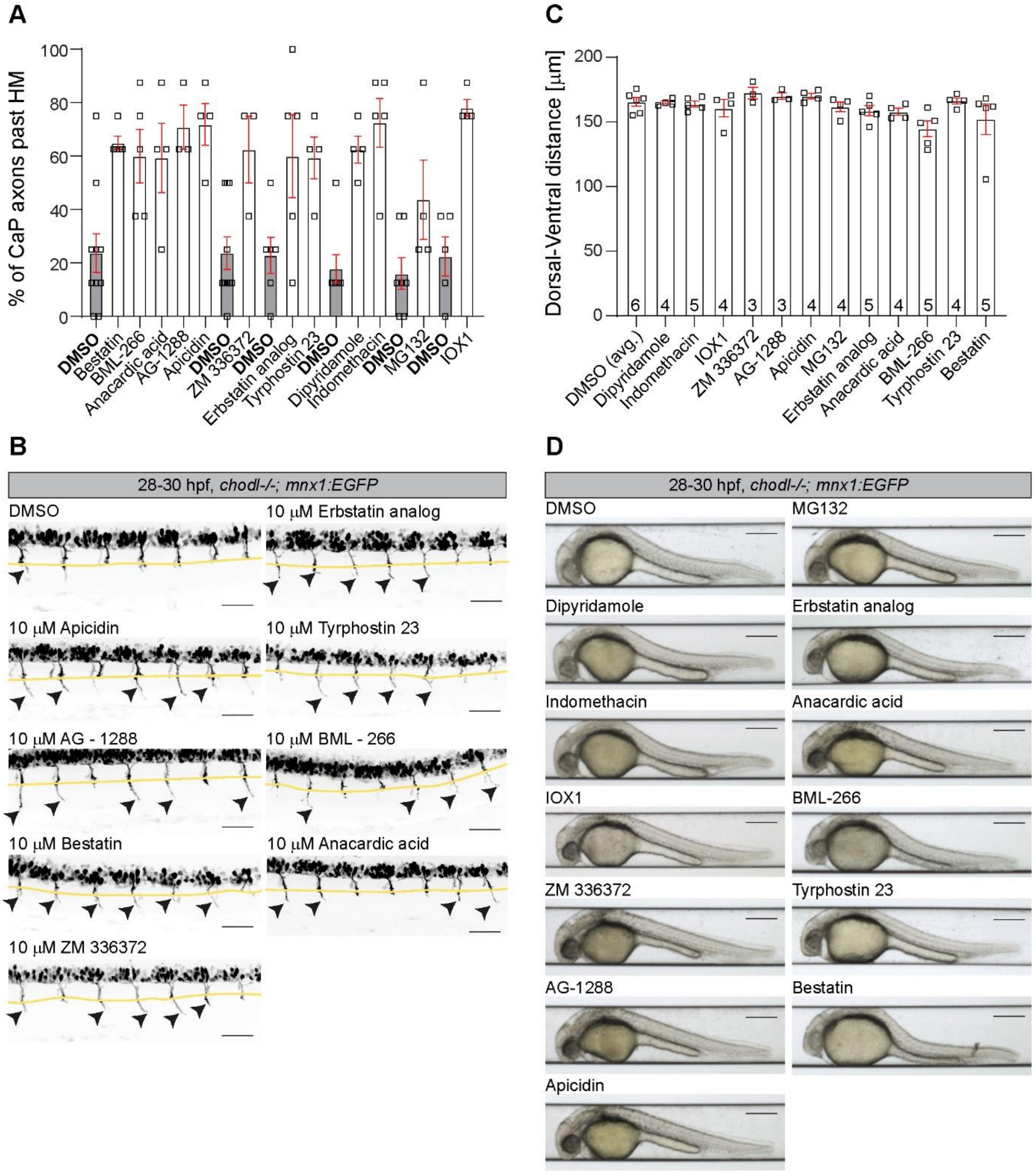

Figure S1: Drug/small molecules from the initial screening that rescue the growth of CaP motor neurons in *chodl* mutant and do not affect the development of the embryos. (A) The percentage of CaP motor axons that pass the horizontal myospetum is shown. Each group is presented in relation to its own DMSO-control (gray bars) preceding each group of drugs. These data relate to Fig. 2B. (B) Representative images of 28-29 hpf embryos (lateral view) after the first screening are shown. Black arrow heads indicate CaP motor axons that passed

beyond the horizontal myoseptum (solid yellow line). Scale bar: 50 µm. (C) Quantification of the length from the dorsal edge of the trunk to the ventral edge of the embryos is shown. No significant differences were observed between DMSO- and drug-treated mutants [Kruskal-Wallis test ($p = 0.0212$) with Dunn's multiple comparison test - no significant differences detected]. (D) Representative images of 28-29 hpf *chodl* embryos that pass through the VAST capillary for imaging are shown. Scale bar: 300 µm.

Each data point represents one animal. N-numbers are indicated in each bar. Error bars represent means ± SEM.

Disease Models & Mechanisms • Supplementary information

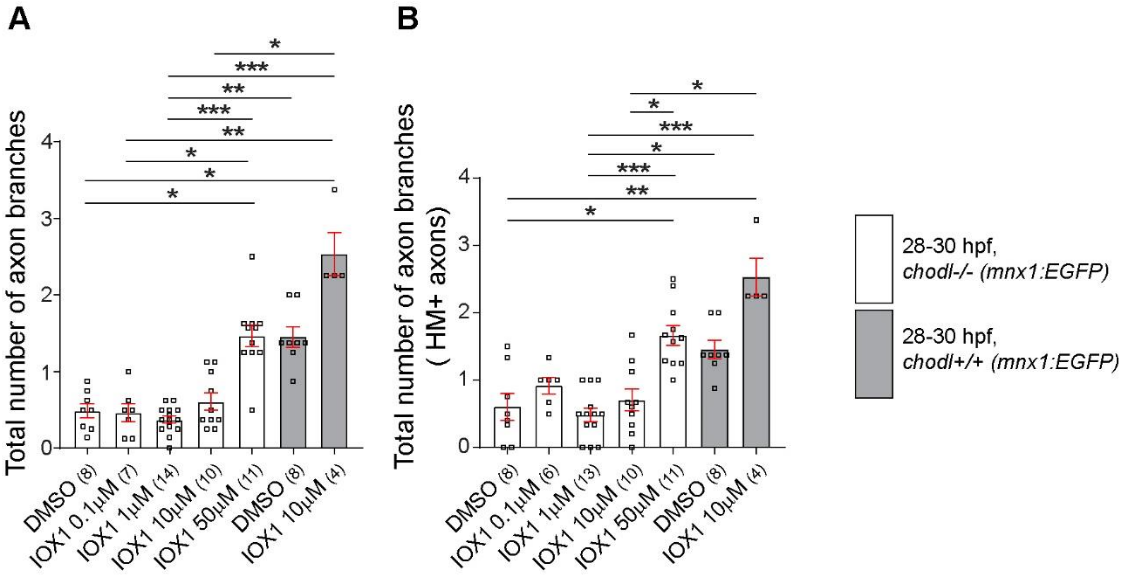

Figure S2: Axonal branching analysis for IOX1-treated *chodl* mutants. (A) Quantification of axonal branching is shown. 50 µm IOX1 induced an increase in the total number of axonal branches, reaching control-levels (Kruskal-Wallis test ****p < 0.0001 with Dunn's multiple comparison test: DMSO-*chodl* mutant vs 50 µM-*chodl* mutant *p = 0.0380, DMSO-*chodl* mutant vs IOX1 10µM-control *p = 0.0131, IOX1 0.1 µM-*chodl* mutant vs IOX1 50 µM-*chodl* mutant *p = 0.0270, IOX1 0.1 µM-*chodl* mutant vs IOX1 10µM-control **p = 0.0093, IOX1 1 µM-*chodl* mutant vs 50 µM-*chodl* mutant ***p = 0.0004, IOX1 1 µM-*chodl* mutant vs DMSO-control **p = 0.0021, IOX1 1 µM-*chodl* mutant vs IOX1 10µM-control ***p = 0.0006, IOX1 10 µM-*chodl* mutant vs IOX1 10µM-control *p = 0.0181, 50 µM-*chodl* mutant vs DMSO-control p > 0.9999, 50 µM-*chodl* mutant vs IOX1 10µM-control p > 0.9999, DMSO-control vs IOX1 10µM-control p > 0.9999, statistical power = 1.00). (B) Quantification of axonal branches for CaP axons that passed the horizontal myoseptum is shown. IOX1 increases the number of branches in *chodl* mutants (Kruskal-Wallis test ****p < 0.0001 with Dunn's multiple comparison test: DMSO-*chodl* mutant vs 50 µM-*chodl* mutant *p= 0.0223, DMSO-*chodl* mutant vs IOX1 10µM-control **p = 0.0092, IOX1 1 µM-*chodl* mutant vs 50 µM-*chodl* mutant ***p = 0.0005, IOX1 1 µM-*chodl* mutant vs DMSO-control *p = 0.0115, IOX1 1 µM-*chodl* mutant vs IOX1 10µM-control ***p = 0.0007, IOX1 10 µM-*chodl* mutant vs IOX1 50 µM-*chodl* mutant *p = 0.0414, IOX1 10 µM-*chodl* mutant vs IOX1 10µM-control *p = 0.0168, 50 µM-*chodl* mutant vs DMSO-control p > 0.9999, 50 µM-*chodl* mutant vs IOX1 10µM-control p > 0.9999, DMSO-control vs IOX1 10µM-control p > 0.9999, statistical power = 1.00).

Each data point represents one animal. N-numbers are indicated in brackets under each bar. Error bars represent means ± SEM.

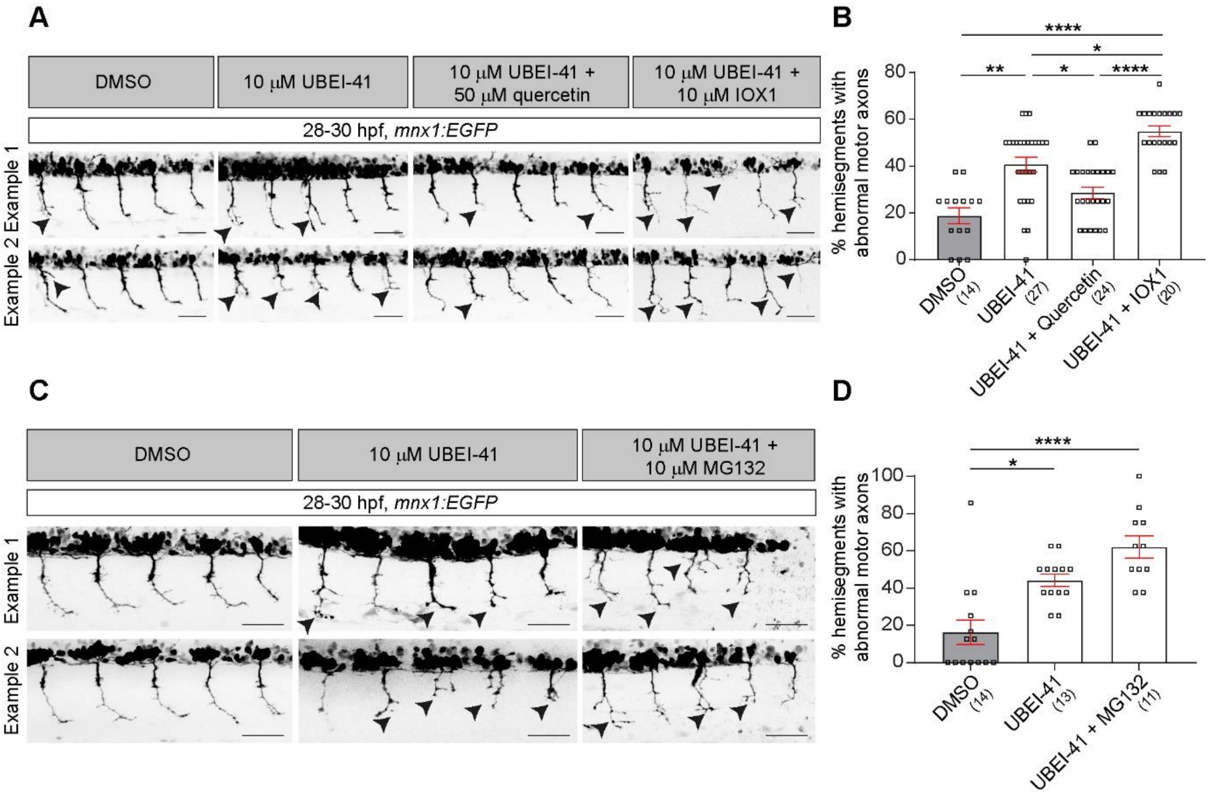

Figure S3: Axonal abnormal morphology of the UBEI-41 model are not rescued by IOX1 or MG132. (A) Representative images (lateral view) of 28-30 hpf drug treated *mnx1:EGFP* embryos (lateral view) are shown: DMSO (vehicle), UBEI-41 (SMA-like phenotype), UBEI-41+ quercetin (internal positive rescue control) and UBEI-41 + IOX1 (experimental condition). (B) The positive control quercetin rescues motor axon morphology induced by UBEI-41 (Kruskal-Wallis test ****p < 0.0001 with Dunn's Multiple Comparison Test: UBEI-41 vs UBEI-41 + quercetin *p = 0.474), but IOX1 treatment does not (DMSO vs UBEI-41 **p = 0.0013, DMSO vs UBEI-41 + IOX1 ****p < 0.0001, UBEI-41 vs UBEI-41 + IOX1 *p = 0.0441). Statistical power for the whole statistical analysis = 0.999. (C) Representative images (lateral view) of 28-30 hpf drug treated *mnx1:EGFP* embryos (lateral view) are shown. (D) Quantification of the abnormal motor axons after MG132 treatment is shown (Kruskal-Wallis test ****p < 0.0001 with Dunn's multiple comparison test: ****p < 0.0001, *p = 0.0139, statistical power = 0.999).

All the examples of abnormal motor neurons are indicated by black arrowheads. All scale bar: 50 µm. Each data point represents one animal. N-numbers are indicated in brackets under each bar. Error bars represent means ± SEM.

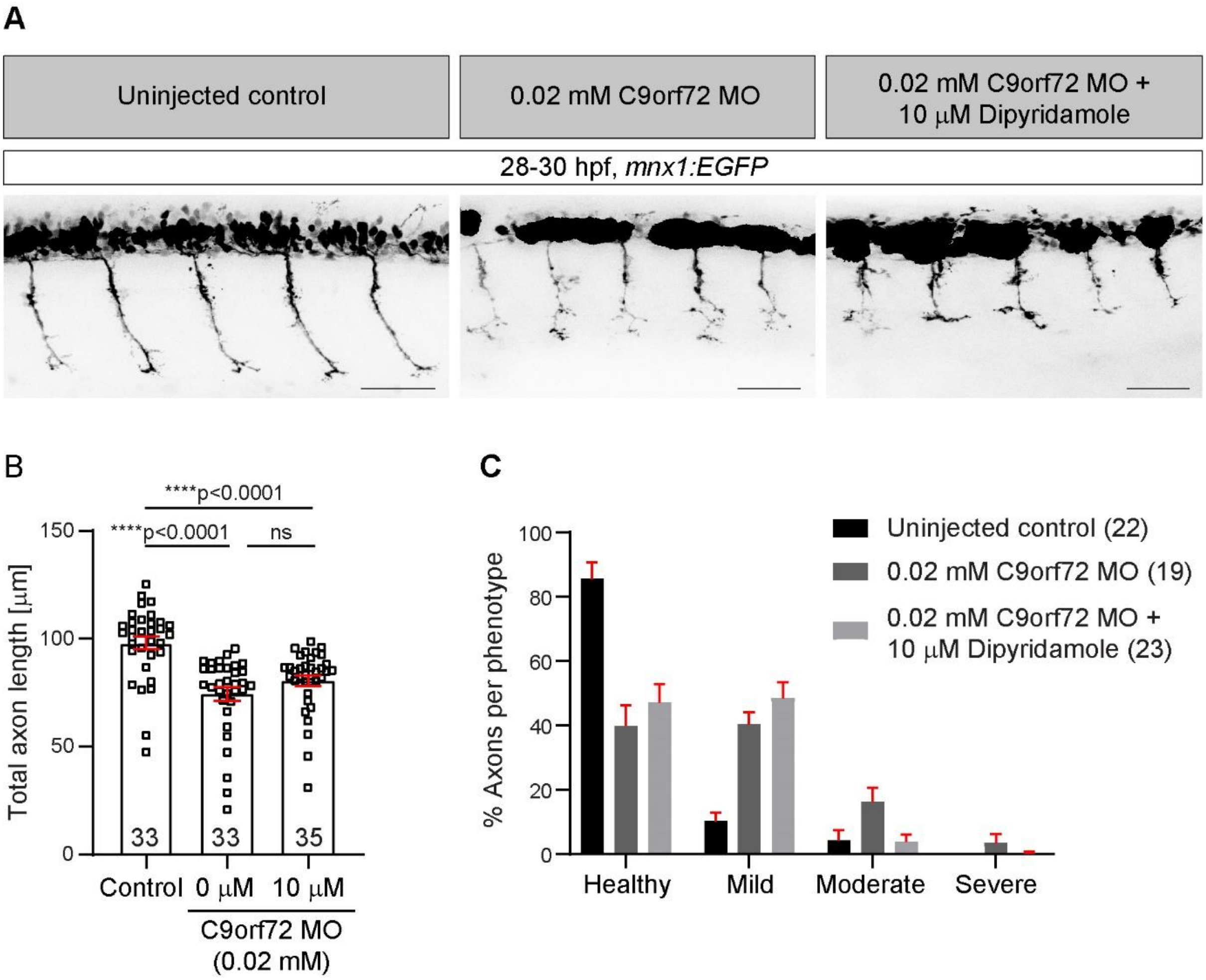

Figure S4: Dipyridamole treatment of C9orf72 knockdown zebrafish does not recue the phenotype. (A) Micrographs of example axons from uninjected controls compared to C9orf72 knockdown without and with 10 µM dipyridamole, respectively. Scale bar: 50 µm. (B) Axon length analysis of C9orf72 knockdown zebrafish treated with dipyridamole. There is no significant difference between axon lengths of dipyridamole-treated zebrafish compared to C9orf72 knockdown alone (Kruskal-Wallis test ****$p < 0.0001$ with Dunn's multiple comparison test, statistical significance power = 0.9998). Each point represents the average axon length of one fish (12 axons per fish). (C) There is no significant difference in axon phenotypes between dipyridamole-treated zebrafish compared to C9orf72 knockdown alone in any category (two-way ANOVA, $p < 0.0001$, $F(6,244) = 23.96$, with Tukey's multiple comparison test, C9orf72 MO vs C9orf72 MO + dipyridamole, 'healthy' $p = 0.3972$; 'mild' $p = 0.3017$; 'moderate' $p = 0.0712$; 'severe' $p = 0.8370$).
N-numbers are indicated in each bar. Error bars represent means ± SEM.
